# Involvement of Interleukin-10 in the Anti-Inflammatory Effect of Sanyinjiao (SP6) Acupuncture in a Mouse Model of Peritonitis

**DOI:** 10.1093/ecam/neq036

**Published:** 2011-06-05

**Authors:** Morgana Duarte da Silva, Giselle Guginski, Maria Fernanda de Paula Werner, Cristiane Hatsuko Baggio, Rodrigo Marcon, Adair Roberto Soares Santos

**Affiliations:** ^1^Departamento de Ciências Fisiológicas, Universidade Federal de Santa Catarina, Campus Universitário, Trindade, 88040-900, Florianópolis, SC, Brazil; ^2^Departamento de Farmacologia, Centro de Ciências Biológicas, Universidade Federal de Santa Catarina, Campus Universitário, Trindade, Florianópolis, SC, Brazil; ^3^Departamento de Farmacologia, Setor de Ciências Biológicas, Universidade Federal do Paraná, Curitiba, PR, Brazil

## Abstract

In this study, we determined the anti-inflammatory effect of manual acupuncture at the Sanyinjiao or Spleen 6 (SP6) point on carrageenan-induced peritonitis in mice and investigated mechanisms that may underlie this effect. In the first set of experiments, male Swiss mice were allocated into five groups: the control (sterile saline), dexamethasone (DEXA), invasive sham-acupuncture (non-acupoint), SP6 acupuncture and carrageenan-treated groups. Ten minutes after needle retention or 30 min after DEXA treatment, mice received an intraperitoneal injection of carrageenan (750 *μ*g/mouse). After 4 h, total leukocyte and differential cell counts (neutrophils and mononuclear), myeloperoxidase (MPO) activity, vascular permeability and cytokine levels were evaluated. In another set of experiments, adrenalectomized (ADX) mice were used to study the involvement of the adrenal gland on the therapeutic effects of acupuncture. Mice were allocated into two groups: the ADX and sham-operated animals (Sham ADX) that were subdivided into four subgroups each: the control (sterile saline), DEXA, SP6 acupuncture and carrageenan-treated groups. The SP6 and DEXA treatments inhibited the inflammatory cell infiltration, vascular permeability and MPO activity in carrageenan-injected mice. In addition, the SP6 treatment also increased interleukin (IL)-10 levels. In contrast, when the animals were adrenalectomized, the SP6 treatment failed to reduce total leukocyte and the plasma extravasation. In conclusion, this study clearly demonstrates the anti-inflammatory effect of SP6 acupuncture in a model of carrageenan-induced peritonitis. Our results demonstrated that SP6 acupuncture depends of the adrenal glands and increased IL-10 levels to produce its anti-inflammatory action.

## 1. Introduction

Acupuncture, a therapeutic modality with few or no adverse effects, is a non-pharmacological therapy in which needles are inserted at specific cutaneous locations of the body, known as acupoints, for the treatment or prevention of several inflammatory diseases, including asthma, rhinitis, inflammatory bowel disease and rheumatoid arthritis [1]. Experimental and clinical trials have shown that acupuncture and electroacupuncture have beneficial effects in painful inflammatory conditions [2, 3]. Despite several studies claiming the success of acupuncture in the treatment of inflammatory disorders, the use of inflammatory/infectious animal models is essential for validating and increasing the knowledge of mechanisms involved in producing the effects of acupuncture therapy.

The Sanyinjiao or SP6 (Spleen 6) acupoint is a spot in the spleen channel that also functions as an important, general tonification point indicated in many disorders, including gynecological, genitourinary, allergic, insomnia, immunological and psychosomatic diseases and pain control [4, 5]. For instance, acupuncture at SP6 has been shown to reduce acetic-acid-induced visceral nociception, and inhibit glutamate-induced nociception, as well as both neurogenic and inflammatory nociceptive responses induced by injection of formalin in mice, according to data observed in our laboratory.

Several studies have been conducted to bring scientific rigor in understanding the physiological mechanisms that support the efficacy of acupuncture [6]. The mechanisms underlying the anti-inflammatory effect of SP6 acupuncture stimulation certainly involve a number of different systems, including activation of the autonomic nervous system, the neuroimmune system and the neuroendocrine system [7, 8]. In this study, we observed the influence of manual acupuncture on peritonitis. Peritonitis is defined as inflammation of the peritoneum, from any cause, that spreads throughout the abdomen and might involve many types of cells, including resident macrophages, which play a critically important role in the orchestration of neutrophil recruitment to the peritoneal cavity [9, 10]. Thus, for this purpose, we evaluated the effect of SP6 acupuncture on an experimental animal model of carrageenan-induced peritonitis and also investigated mechanisms responsible for its anti-inflammatory effects.

## 2. Methods

### 2.1. Animals

Experiments were conducted using adult male Swiss mice weighing 25–35 g, housed at 22°C under a 12-h light/12-h dark cycle (lights on at 06:00 h) and with access to food and water *ad libitum*. They were acclimatized to the laboratory for at least 1 h before use. All experiments were previously approved by Universidade Federal de Santa Catarina Committee on the Ethical Use of Animals and were carried out in accordance with the international standards and the ethical guidelines on animal welfare.

### 2.2. Acupuncture Treatment Procedures

**Figure 1:**
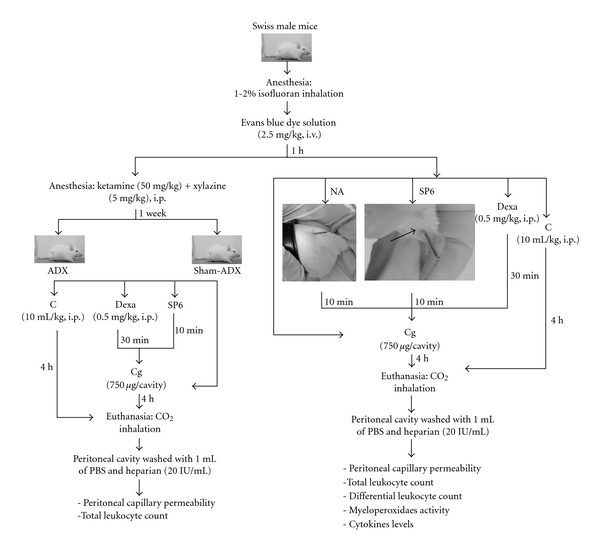
A flow diagram of this study.

For the study, animals were randomized in two groups: the SP6 acupuncture and invasive sham acupuncture (non-acupoint) groups. Mice were gently handled and lightly restrained in a plastic cylinder (7 × 2.5 cm) with the right hind limb out of the tube for needling. After cleaning the skin with alcohol, manual acupuncture stimulation was performed by obliquely inserting a stainless steel needle (0.17 × 7 mm) to a depth of about 2-3 mm at right Sanyinjiao (SP6) and the needle was then rotated slowly. The entire procedure was completed in less than 15 s. In mice, the SP6 acupoint is located 2 mm proximal to the upper border of medial malleolus, between the posterior border of the tibia and the anterior border of the Achilles tendon [11]. For assessment of the specific effects triggered by SP6 acupuncture stimulation, another group of mice were punctured at a non-acupuncture point. For the sham-treated control, the needle was inserted 5 mm lateral to the midline of the posterior surface of the hind limb, based on the non-acupoint in rat [12]. After inserting the needle, each mouse was placed in a transparent acrylic box (10 × 10 × 10 cm^3^) for the entire 10-min treatment. The animals remained awake, and no signs of distress were observed during acupuncture stimulation (Figure 1).

### 2.3. Carrageenan-Induced Peritonitis

The carrageenan (no treatment) group, the SP6 acupuncture group and the invasive sham acupuncture (non-acupoint) group (immediately after the end of acupuncture treatments) received an intraperitoneal (i.p.) injection of 0.5 mL of carrageenan (750 *μ*g per cavity) diluted in sterile saline according to procedures described previously [13]. Using carrageenan as a stimulus, it was possible to produce an acute inflammatory response after 4 h in the peritoneal cavity of mice, with a large number of leukocytes in the exudates. The effect was reversed by glucocorticoid treatment.

The positive control group was pre-treated with dexamethasone (DEXA) (0.5 mg kg^−1^, i.p.) 30 min before carrageenan injection. The synthetic glucocorticoid DEXA is the most effective steroidal antiinflammatory treatment available for several allergic and inflammatory diseases; and its dose was chosen according to data in the literature [14] and also based on previous studies of our laboratory. The control group received a similar volume of vehicle (sterile saline, 10 mL kg^−1^, i.p.). In short, the groups formed were as follows: the control group (C) with sterile saline i.p. (1a), the carrageenan i.p. group (2a), the DEXA plus carrageenan i.p. group (3a), the sham acupuncture (NA) plus carrageenan i.p. group (4a) and the SP6 acupuncture (SP6) plus carrageenan i.p. group (5a). Four hours after peritonitis induction, mice were sacrificed by CO_2_ asphyxiation, according to guidelines for the care and use of experimental animals [15]. The peritoneal fluid was collected for further analysis (Figure 1).

### 2.4. Peritoneal Leukocyte Counts

The peritoneal cavity was opened and washed with 1 mL of sterile phosphate buffered saline (PBS) containing heparin (20 IU mL^−1^). Total leukocyte counts were performed in a Neubauer chamber after diluting the peritoneal fluid with Türk solution (1:20). Peritoneal cells were cytocentrifuged onto slides using a Cytospin (Tharmac, Germany) and stained with May-Grünwald Giemsa to determine the differential leukocyte count [14].

### 2.5. Peritoneal Capillary Permeability

In the beginning of the experiment, mice were anesthetized by isoflurane inhalation (1-2%) and Evans blue dye solution (25 mg kg^−1^), used as peritoneal capillary permeability marker, was injected intravenously. A sample of the fluid collected (500 *μ*L) from the peritoneal space was separated and stored at –20°C to determine the concentration of Evans blue dye. The amount of extravasated Evans blue was measured spectrophotometrically at 620 nm. The peritoneal capillary permeability induced by carrageenan was expressed in terms of the concentration of the dye (*μ*g mL^−1^) that leaked into the peritoneal cavity by interpolation from the standard curve of Evans blue in the range of 5–100 *μ*g mL^−1^.

### 2.6. Peritoneal Fluid MPO Assay

MPO activity, an indicator of neutrophil accumulation, in peritoneal fluid was assessed 4 h after peritonitis was induced with carrageenan in mice [14, 16]. The exudates were centrifuged at 20 000 g for 30 min at 4°C. An aliquot was then allowed to react with a solution of 1.6 mM tetramethylbenzidine HCl in dimethylformamide and 0.1 mM hydrogen peroxide in 96-well plates. Plates were incubated at 37°C for 3 min, and then the reaction was stopped by the addition sodium acetate (1.46 M, pH 3.0). MPO activity was estimated by means of colorimetric measurements using a plate reader (BMG Labtec, Germany) set to measure absorbance at 650 nm and expressed as DO/mLml. Samples of peritoneal fluid from control and acupuncture-treated animals were collected and immediately processed for analysis of MPO levels.

### 2.7. Determination of Cytokines Levels in Peritoneal Fluid

Four hours after carrageenan-induced peritonitis occurred, peritoneal fluid from the treated mice was used to estimate the cytokine levels by enzyme-linked immunosorbent assay (ELISA) [17]. Sample aliquots of 100 *μ*L were used to measure their tumor necrosis factor alpha (TNF-*α*), interleukin (IL)-1*β* and IL-10 levels using mouse cytokine ELISA kits from R&D Systems (Minneapolis, MN), according to the manufacturer's instructions. The absorbance for all cytokines studied was measured using a microplate reader at 450 and 550 nm.

### 2.8. Involvement of Adrenal Glands

In order to investigate the participation of the adrenal glands in the anti-inflammatory activity of SP6 acupuncture, mice were anesthetized with ketamine (50 mg kg^−1^, i.p.) and xylazine (5 mg kg^−1^, i.p.) and the bilateral adrenalectomy (ADX) was performed through a dorsal incision, as previously described [18]. Sham-operated animals were submitted to the same procedure without removing the adrenal glands. After surgery, the animals were returned to their cages, with free access to food and drink, but water was replaced by saline (0.9% NaCl solution) in ADX mice to maintain a physiologically relevant plasma sodium concentration. After 1 week, the animals were treated with SP6, non-acupuncture (sham) or DEXA (0.5 mg kg^−1^, i.p.). Control animals received a similar volume of the appropriate vehicle (10 mL kg^−1^, i.p.). In summary, the groups included sham-operated (Sham ADX): control (1b), carrageenan treated (2b), DEXA-treated (3b) and SP6 groups (4b); the operated animals (ADX) belonged to the control group (C) with sterile saline i.p. (1c), the carrageenan i.p. group (2c), the DEXA plus carrageenan i.p. group (3c) or the SP6 acupuncture (SP6) plus carrageenan i.p. group (4c). The animals were sacrificed by CO_2_ asphyxiation 4 h after injection of carrageenan, and peritonitis was then evaluated.

### 2.9. Drugs and Reagents

The following substances were used: carrageenan (Sigma Chemical Co., St. Louis, MO, USA), DEXA (Aché Laboratórios Farmacêuticos S/A, Brazil), ketamine (Vetbrands Limited, Brazil); xylazine (Carlier S.A., Barcelona, Spain). Cytokines levels were evaluated using ELISA kits from R&D Systems. Drugs were dissolved in saline solution.

### 2.10. Statistical Analysis

Data were expressed as mean ± SEM and were statistically evaluated by one-way ANOVA followed by the post-hoc Student-Newman-Keuls test using GraphPad Software (San Diego, CA). The significance level in all cases was set at *P* < .05.

## 3. Results

### 3.1. SP6 Acupuncture Reduces Carrageenan-Induced Peritonitis in Mice

**Figure 2:**
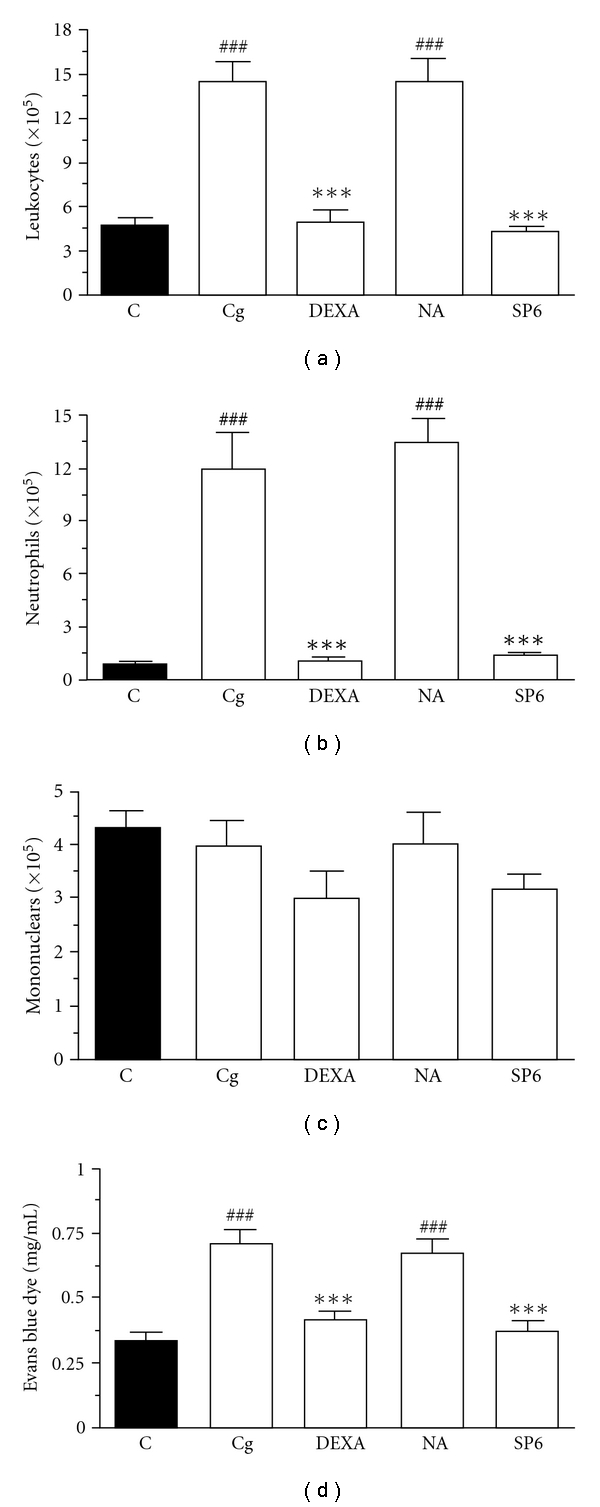
Effect of SP6 acupuncture or DEXA in carrageenan-induced peritonitis in mice. (a) Total leukocytes, (b) neutrophil cells, (c) mononuclear cells and (d) Evans blue content (exudation). Mice received sterile saline (NaCl, 0.9%, i.p., C), carrageenan (750 *μ*g/mice, Cg), DEXA (0.5 mg kg^−1^, i.p., 0.5 h, DEXA) plus Cg, stimulation of the sham acupoint (10 min, NA) plus Cg or stimulation of SP6 acupoint (10 min, SP6) plus Cg. Values are mean ± SEM of six to eight animals. ***P < .001 compared with carrageenan groups, and ^###^ *P* < .001 compared to control group (one-way ANOVA followed by Newman-Keul's test).

As ascertained before, the injection of carrageenan resulted in significant increases in both leukocyte numbers and exudation into the peritoneal cavity (Figures 2(a)–2(d)). Thus, following carrageenan administration, a gradual enhancement in fluid leakage and in the total number of cells that migrate into the peritoneal space was observed, due primarily to neutrophils. However, the number of peritoneal mononuclear cells was not modified by carrageenan injection (Figure 2(c)).

SP6 acupuncture treatment performed before carrageenan injection promoted significant inhibition of the inflammatory process due to carrageenan-induced peritonitis. Our results showed a complete decrease in total cell migration (*P* < .001) (Figure 2(a)), represented mainly by neutrophil influx with inhibition of 96 ± 2% (*P* < .001) (Figure 2). Moreover, the treatment of mice with the steroidal anti-inflammatory DEXA (0.5 mg kg^−1^, i.p.) also completely decreased total cell migration, represented mainly by neutrophil influx with inhibition of 100% (*P* < .001; Figures 2(a) and 2(b)).

The peritoneal inflammation induced by carrageenan was accompanied by an increase in abdominal vascular permeability, observed by Evans blue dye exudation. Our results indicated that both SP6 acupuncture and systemic DEXA treatment significantly reduced Evans blue extravasation by 95 ± 4 and 96 ± 5%, respectively (Figure 2(d)).

**Figure 3:**
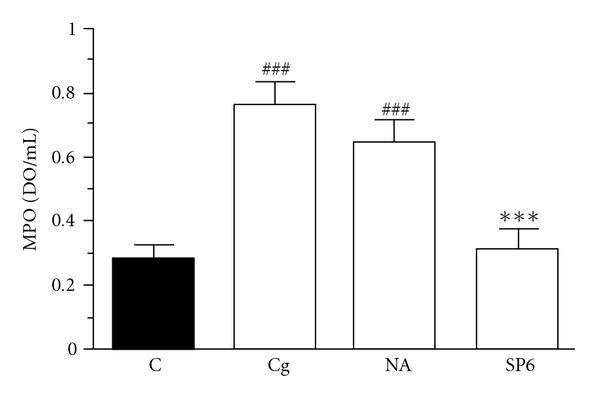
Effect of SP6 acupuncture on MPO activity in peritoneal fluid from carrageenan-induced peritonitis in mice. Mice received sterile saline (NaCl, 0.9%, i.p., C), carrageenan (750 *μ*g/mice, Cg), stimulation of the sham acupoint (10 min, NA) plus Cg or stimulation of SP6 acupoint (10 min, SP6) plus Cg. Each column represents the mean ± SEM of four to five animals. ***P < .001 compared with the carrageenan group, and ^###^ *P* < .001 compared to control group (one-way ANOVA followed by Newman-Keul's test).

The neutrophil migration seen in mice with carrageenan-induced peritonitis was also indirectly determined by MPO activity. Thus, treatment of mice with SP6 acupuncture or DEXA also significantly prevented the increase in MPO activity induced by carrageenan, with inhibition of 98 ± 1 and 100%, respectively (Figure 3).

### 3.2. SP6 Acupuncture and TNF-*α*, IL-1*β* and IL-10 Levels in Mice with Carrageenan-Induced Peritonitis

**Figure 4:**
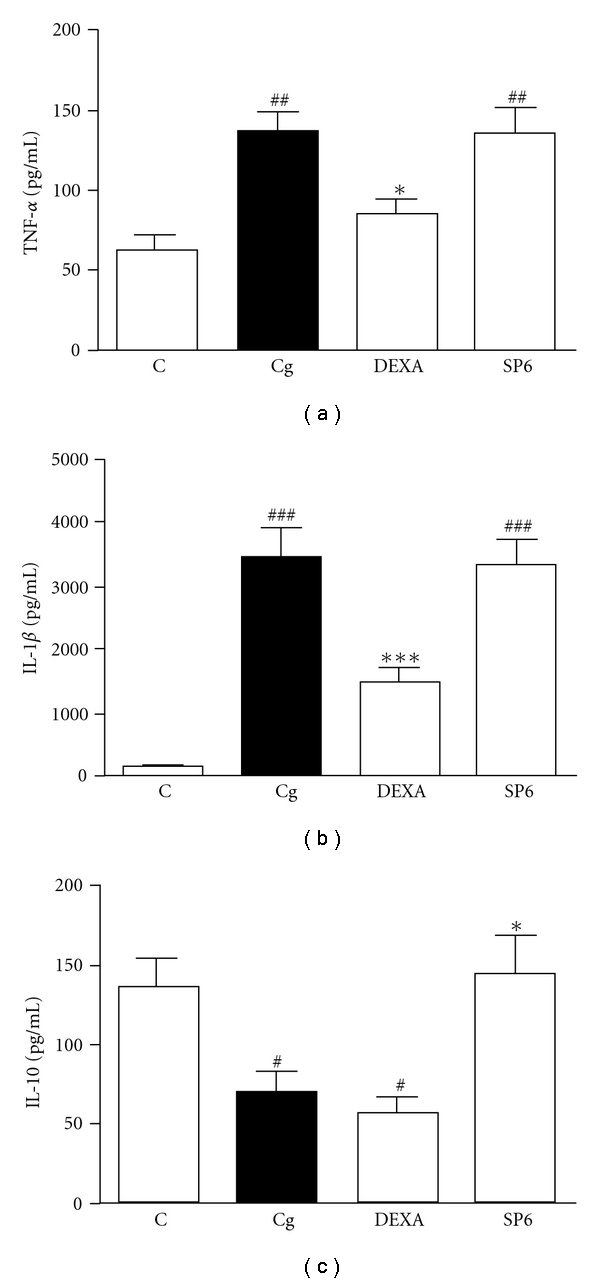
Effect of SP6 acupuncture or DEXA upon TNF-*α* (a), IL-1*β* (b) and IL-10 production (c) in carrageenan-induced peritonitis in mice. Mice received sterile saline (NaCl, 0.9%, i.p., C), carrageenan (750 *μ*g/mice, Cg), DEXA (0.5 mg kg^−1^, i.p., DEXA) plus Cg or stimulation of SP6 acupoint (10 min, SP6) plus Cg. Each group represents the mean of four to five animals and the vertical bars the SEM. *P < .05, ***P < .001 compared with carrageenan groups and ^#^ *P* < .05, ^##^ *P* < .01, ^###^ *P* < .001 compared with carrageenan groups (one-way ANOVA followed by Newman-Keul's test).

Our results demonstrated that SP6 acupuncture treatment was effective in decreasing the cell migration and exudation in carrageenan-induced peritonitis. In this study, the mechanisms involved in SP6 acupuncture-induced anti-inflammatory effects were evaluated. Figures 4(a)–4(c) shows that TNF-*α*, IL-1*β* and IL-10 could be detected in the peritoneal fluid from control groups. However, 4 h after carrageenan injection, TNF-*α* and IL-1*β* levels increased to 137 and 3480 pg mL^−1^, and IL-10 levels decreased to 69 pg mL^−1^, relative to the control groups (62, 192 and 135 pg mL^−1^, resp.).

Furthermore, treatment of the animals with DEXA caused significant inhibition of the TNF-*α* and IL-1*β* levels in peritoneal fluid by 86 and 1489 pg mL^−1^ (Figures 4(a) and 4(b)), respectively, while IL-10 levels were unchanged (Figure 4(c)). In sharp contrast, SP6 acupuncture did not reduce TNF-*α* and IL-1*β* levels in peritoneal fluid (Figures 4(a) and 4(b)), but significantly increased the anti-inflammatory cytokine IL-10 levels (by 143 pg mL^−1^) after carrageenan injection (Figure 4(c)).

### 3.3. Endogenous Glucocorticoids Contributed to the Anti-Inflammatory Effect of SP6 Acupuncture in Carrageenan-Induced Peritonitis

**Figure 5:**
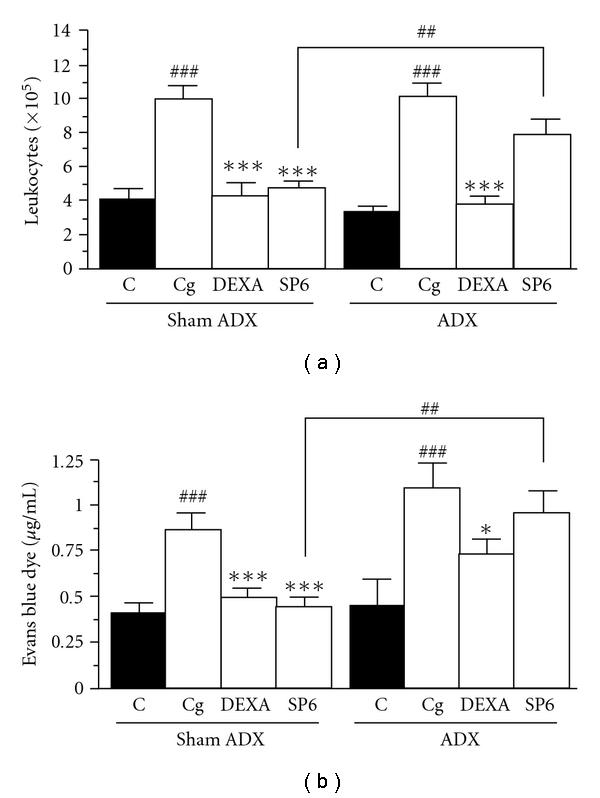
Effect of SP6 acupuncture in carrageenan-induced peritonitis in adrenalectomized mice: number of leukocytes (a) and Evans blue dye extravasation in the peritoneal cavities (b). ADX was performed 7 days before the start of the experiment. Mice received sterile saline (NaCl, 0.9%, i.p., C), carrageenan (750 *μ*g/mice, Cg), DEXA (0.5 mg kg^−1^, i.p., 0.5 h, DEXA) plus Cg or stimulation of SP6 acupoint (10 min, SP6) plus Cg. Values are mean ± SEM of six to eight animals. *P < .05, ***P < .001 when compared with carrageenan group, ^###^ *P* < .001 when compared to control and SP6 sham ADX group, ^##^ *P* < .01 when compared between Sham ADX SP6 and ADX SP6 groups (one-way ANOVA followed by Newman-Keul's test).

As shown in Figure 5(a), there was a significant difference in the number of migrating leukocytes in the peritoneal fluid of carrageenan-injected animals in comparison to controls of sham-operated mice and of ADX mice. However, the number of migrating leukocytes in ADX mice was similar to sham-operated mice after the carrageenan injection.

The treatments with DEXA reduced the number of migrating leukocytes in ADX carrageenan-treated animals and in sham-operated animals in 85 ± 7 and 93 ± 3%, respectively. On the contrary, the SP6 acupuncture treatment inhibited the total leukocytes only in the sham-operated mice in 75 ± 9%. No significant inhibition of the cell accumulation was observed with SP6 acupuncture treatment in ADX carrageenan-treated animals (Figure 5(a)). This same pattern was observed in plasma extravasation using Evans Blue (Figure 5(b)).

## 4. Discussion

According to traditional Chinese medicine, overall health depends of formation, maintenance and circulation of Yin and Yang, and their imbalance results in the development of diseases. The inflammatory peritoneal processes promotes a “depletion syndrome" that can show signs of prostation (Qi), asthenia (Yin) and impairment (Yang), as a result of an infection. Thus, management of inflammatory peritoneal conditions using Chinese herbal medicine or acupuncture to treat the “depletion syndrome" could strengthen the immune system against infection by suppressing and/or neutralizing pathogens, as well as through the inhibition of inflammatory mediator production [7, 11, 19, 20]. Importantly, acupuncture is a traditional form of Chinese medicine that has been accepted and used worldwide for its effects on various physiological regulatory mechanisms and control of pathological changes. The SP6 point of “The Spleen Meridian of Foot-Taiyin" is commonly used in human acupuncture to treat a wide range of health conditions, including gastric disorders as stomachache, abdominal pain and distension, constipation, diarrhea, vomiting, dysentery, indigestion, children's autism and others [21–23].

This study was the first to demonstrate that unilateral manual acupuncture at the SP6 point, without electrical stimulation, elicited pronounced anti-inflammatory actions in mice submitted to carrageenan-induced peritonitis, a model of acute inflammation. In addition, this study provides the first assessment of the ability of the SP6 acupoint to markedly inhibit leukocyte infiltration, abdominal vascular permeability, MPO activity and increase IL-10 levels in peritoneal fluid, as well as the possible contribution of endogenous glucocorticoids to these anti-inflammatory effects.

Acute inflammation involves microvascular changes with increased vascular permeability, flow of exudation, including plasmatic protein, cell migration (primarily neutrophils) and amplification of endogenous chemical mediators into the site of injury [24]. Among experimental models, carrageenan-induced peritonitis is a well-characterized experimental model of acute inflammation, largely employed to test new anti-inflammatory therapies that permit the quantification and correlation of both exudates and cellular migration with changes in other inflammatory parameters [25–27]. However, there is currently no data demonstrating definitive usefulness of SP6 acupuncture in peritonitis models. When acute peritoneal inflammation was induced in mice by carrageenan, SP6 acupuncture reduced leukocyte influx, predominantly of neutrophils, 4 h after peritonitis induction. Assessment of the peritoneal fluid also revealed that SP6 acupuncture reduced the peritoneal leakage and MPO activity (a marker of neutrophil content), another important feature of this inflammatory model. These findings corroborate previous reports that acupuncture, performed at other acupoints such as Yintang (HN3), Houhai or Changqiang (GV1), Baihui (GV20) and Zusanli (ST36), respectively, produces anti-inflammatory effects on carrageenan-induced peritonitis and sepsis induced by cecal ligation in rats [1, 28]. In fact, this group reported that acupuncture inhibited neutrophil migration and partially re-established neutrophil migration into the peritoneal cavity after peritonitis induced by carrageenan and sepsis, respectively. We believe that the anti-inflammatory effect observed in our study is strongly associated with the specific SP6 stimulation point, since sham-acupuncture did not affect cell migration. Furthermore, acute, unilateral, manual SP6 acupuncture exhibited longer-lasting anti-inflammatory effects, since 4 h was the time span for the acupuncture treatment, carrageenan injection and termination of the experiment. Acupuncture has been used to treat immune diseases, like asthma, using acupuncture points that boost the vital energy and regulate the immune system [29]. In a model of collagen-induced arthritis (CIA), manual acupuncture was less effective than eletroacupuncture at the ST36 acupoint in treated, arthritic animals, reinforcing that needle retention can be beneficial to the relief or treatment of CIA, but supplemental electrical stimulation intensifies its effects [2]. In addition, here, mice were not anesthetized during SP6 stimulation, reinforcing that therapeutic effects observed did not involve nonspecific effects of needling or stress.

A number of pro-inflammatory mediators are involved in the acute inflammation induced by carrageenan, such as neuropeptides, prostaglandins, nitric oxide and cytokines [30]. Regarding the participation of pro-inflammatory cytokines in this model, it has been clearly established that leukocytes, among other cells, produce IL-1*β*, IL-6, IL-8 and TNF-*α* [31]. On the other hand, macrophages produce IL-10, an anti-inflammatory cytokine that plays an important role in control of inflammation [30]. Of note, mice submitted to carrageenan-induced peritonitis exhibited increased IL-1*β* and TNF-*α* levels, in contrast to reduced IL-10 levels in peritoneal fluid. The SP6 acupuncture did not modify IL-1*β* and TNF-*α* levels in peritoneal fluid, but increased IL-10 levels. Scognamillo-Szabó et al. [28] showed that acupuncture performed using a combination of the acupoints Yintang, Houhai and Baihui reduced IL-1*β* but did not change TNF-*α* and IL-10 peritoneal levels in the carrageenan-induced peritonitis model.

The reasons for the discrepant findings are still unclear, but the type of acupuncture stimulation (specific acupoint) and animal species involved might account for the observed differences. At this time, it is possible that the mechanism for the anti-inflammatory effect of SP6 acupuncture could be attributed to the increase in IL-10 release. IL-10 has received much attention because of its anti-inflammatory properties. Uniquely, among hematopoietic cytokines, IL-10 is a pleiotropic molecule that displays both immunostimulatory and immunoregulatory activities [32].

Additionally, our data demonstrated that DEXA, a synthetic glucocorticoid with potent anti-inflammatory and immunosuppressant properties; also inhibited leukocyte and neutrophil influx, abdominal vascular permeability and MPO activity in the peritoneal fluid of carrageenan-injected mice. However, DEXA reduced the pro-inflammatory cytokines (IL-1*β* and TNF-*α*) but did not increase IL-10 levels in peritoneal fluid of mice with peritonitis. Therefore, our studies suggest that the anti-inflammatory effects of SP6 acupuncture and DEXA might be due to different mechanisms.

Another important, novel finding of this study was the demonstration that ADX was able to significantly reverse the anti-inflammatory action of SP6 acupuncture, but not the anti-inflammatory action produced by DEXA in carrageenan-induced peritonitis. These data suggested that SP6 acupuncture activated the adrenal glands, subsequently regulating carrageenan-induced peritonitis. Additionally, by the same mechanism, electroacupuncture increased plasmatic endogenous glucocorticoids, suppressing the edema in the paw of complete Freund's adjuvant-inflamed rats [33–35].

A variety of studies suggested that inflammatory information is transmitted through sensory nerves to the hypothalamus, where input signals are processed; it then results in an anti-inflammatory output via the autonomic nervous system. The thought that acupuncture might be involved as a modulator of the immune system has recently been supported by several observations, and it has been suspected that acupuncture might affect immune modulation [36–38]. Although actual scientific evidence is yet to be produced, studies relating to neuroimmunology [39, 40] and autonomic reflexes could form a significant base for understanding the basic acupuncture mechanism as a neural-immune reflex [38]. In addition, transcutaneous electrical stimulation of the ST36 and SP6 acupoints was effective in reducing the percentage of body fat and waist circumference in postmenopausal women. The authors suggested that this effect might be due to modulation of the autonomic nervous system [41].

Pro-inflammatory cytokines are involved in the interaction between the brain and immune system, stimulating neural outflows via the autonomic nervous system [38, 40]; parasympathetic nerve endings release acetylcholine, and a neuroimmune reflex appears to suppress the release of inflammatory cytokines [39, 40]. This theory could explain the anti-inflammatory effect of acupuncture, reducing cell migration and edema caused by peritonitis induced by carrageenan. However, acupuncture might inhibit the synthesis of pro-inflammatory cytokines and, in our study, SP6 acupuncture did not change IL-1*β* and TNF-*α* levels, but increased IL-10 levels in peritoneal exudates.

**Figure 6:**
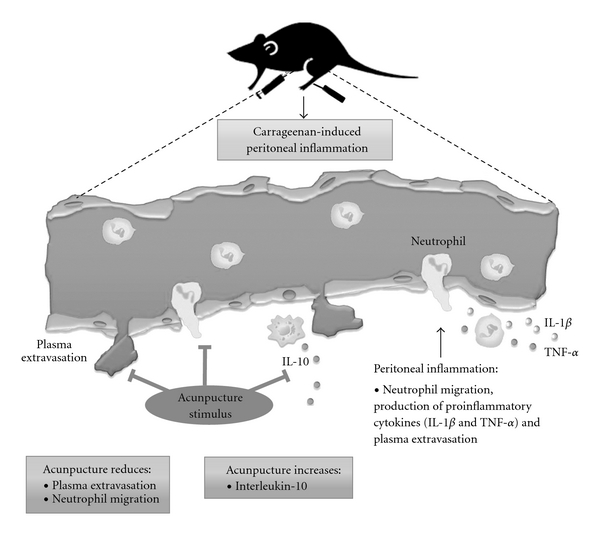
SP6 acupuncture activated the adrenal glands and increased the IL-10 levels in the regulation of carrageenan-induced peritonitis. Carrageenan produces an acute inflammatory response after 4 h in the peritoneal cavity of mice, with a large migration of leukocytes into the exudates, plasmatic extravasation and increase in inflammatory cytokine levels (TNF-*α* and IL-1*β*). SP6 acupuncture elicited pronounced anti-inflammatory effects by inhibition of leukocyte infiltration and abdominal vascular permeability, and increased IL-10 levels in peritoneal fluid.

In summary, this study was the first to demonstrate, that unilateral SP6 acupuncture without electrical stimulation elicited significant anti-inflammatory effects in a mouse model of peritonitis caused by carrageenan. Our data demonstrated that the anti-inflammatory effects of SP6 acupuncture depended on the adrenal glands and increased IL-10 levels (Figure 6). Further investigation is required to completely elucidate the mechanisms underlying the anti-inflammatory effect of SP6 acupuncture.
